# A case report of acute heart failure, pulmonary edema, and pleural effusion in an elderly patient with organic mental disorder: An integrative holistic nursing approach

**DOI:** 10.1097/MD.0000000000050410

**Published:** 2026-08-28

**Authors:** Guohui Jiao, Yongcai Wu, Shanshan Zhang, Haiyan Wang, Xiaoli Du

**Affiliations:** a Inner Mongolia Autonomous Region Mental Health Center (The Third Hospital of Inner Mongolia Autonomous Region, Inner Mongolia Autonomous Region Brain Hospital), Hohhot, Inner Mongolia, China; b Baotou Teachers’ College, Baotou, Inner Mongolia, China

**Keywords:** acute heart failure, holistic nursing care, organic mental disorder, organic mental disorder, pleural effusion, pleural effusion, pulmonary edema, pulmonary edema

## Abstract

**Rationale:**

Organic mental disorders complicate acute heart failure (AHF) in elderly patients, creating a heart–brain vicious cycle in which cognitive impairment masks cardiac symptoms while hypoxemia worsens delirium. Holistic nursing offers an integrative solution to this multidimensional complexity.

**Patient concerns:**

A 67-year-old male with a 5-year history of visual hallucinations, insomnia, and poorly controlled hypertension presented with generalized edema and severe dyspnea. On admission, oxygen saturation was 60% (85% with oxygen), blood pressure was 139/90 mm Hg, and bedside ultrasound revealed bilateral pleural effusions (86 mm and 122 mm).

**Diagnosis:**

Organic mental disorder, AHF (New York Heart Association class IV), bilateral pleural effusion, pulmonary edema. complete left bundle branch block, hypertension stage 2, hepatic impairment, hypoproteinemia, and hypochloremia.

**Interventions:**

Holistic nursing included continuous triad assessment of consciousness, respiration, and circulation; low-flow oxygen and semi-recumbent positioning; nitroglycerin and furosemide with dynamic adjustment; ultrasound-guided thoracentesis with 660 mL drainage; daytime cognitive orientation; nighttime 3 mg zopiclone; precise volume management maintaining 400 to 500 mL negative daily balance; high-protein nutrition; and bundled infection control.

**Outcomes:**

After 16 days, dyspnea and edema resolved, pleural effusions cleared, and ejection fraction improved. Orientation was restored, weight fell from 65 kg to 53.8 kg, and no nosocomial infections occurred. At 3-month follow-up, vitals were stable, the patient was adherent to medications and self-sufficient, with no recurrence of AHF or psychiatric symptoms.

**Lessons:**

Holistic nursing effectively manages cardiopulmonary-psychiatric comorbidities through multidimensional assessment and multidisciplinary collaboration. Strengthening discharge planning and hospital-community continuity is essential.

## 1. Introduction

Organic mental disorders constitute a group of mental disorders directly caused by organic brain lesions or somatic diseases, with an incidence rate as high as 30 to 40% among elderly patients with somatic diseases.^[1]^ Acute heart failure (AHF), a common critical emergency in the elderly, forms a vicious cycle of heart–brain interaction when combined with organic mental disorders. Cognitive impairment masks early symptoms of heart failure, delaying treatment, while hypoxemia and cerebral hypoperfusion caused by heart failure exacerbate psychiatric symptoms.^[2]^ This comorbidity significantly increases readmission rates and mortality, posing substantial challenges to clinical care.^[3]^

Patients with organic mental disorders often experience hallucinations, disorientation, and agitation, making it difficult for them to accurately express physical discomfort and potentially masking early signs of AHF. Concurrently, dyspnea and sleep deprivation from AHF exacerbate delirium and increase myocardial oxygen consumption, creating a vicious cycle of deterioration.^[4]^ Additionally, these patients often present with pulmonary edema and pleural effusion, coupled with poor treatment adherence, rendering traditional single-specialty care models inadequate for managing such multidimensional complexity.

Holistic nursing emphasizes viewing individuals as unified wholes encompassing physiological, psychological, social, and spiritual dimensions. Through multidimensional assessment and individualized interventions, it aims to achieve whole-person care.^[5]^ Research indicates that holistic nursing-based interventions significantly improve the physical and psychological status of heart failure patients. By establishing integrated care models through multidisciplinary collaboration, these approaches effectively reduce readmission rates for cardiovascular events in patients with cognitive impairment.^[6]^ Currently, nursing research addressing acute cardiopulmonary diseases co-occurring with organic mental disorders remains insufficient. This case report aims to provide a reference for clinical nurses managing such complex critical patients by summarizing holistic nursing practices applied to an elderly patient with organic mental disorder complicated by AHF, pulmonary edema, and pleural effusion.

## 2. Medical record

### 2.1. Clinical manifestations

Written informed consent was obtained from the patient for the publication of this case report. Patient: male, 67 years old, height 170 cm, weight 65 kg. Presented to a local hospital with a 5-year history of poor sleep, visual hallucinations, insomnia, emotional instability, incoherent speech, generalized edema, and unsteady gait with shortness of breath. After discharge, worsening symptoms led to his transfer to our hospital for treatment. On admission, the patient’s temperature was 36.5°C, blood pressure 139/90 mm Hg. He had a 5-year history of hypertension with peak readings of 160/100 mm Hg. He irregularly took amlodipine besylate with inadequate blood pressure control. Partial pressure of arterial oxygen was 60% (85% after oxygen administration). Thyroid-stimulating hormone: high-sensitivity troponin I: 114.60 pg/mL; myoglobin: 574.30 ng/mL; creatine kinase isoenzyme: 38.80 ng/mL; activated partial thromboplastin time: 15.90 s; international normalized ratio: 1.43. Serum biochemistry: alanine aminotransferase: 157.40 U/L; aspartate aminotransferase (AST): 223.00 U/L; total protein: 48.30 g/L; total bilirubin: 24.20 μmol/L; alkaline phosphatase: 160.60 U/L; urea: 13.0 mmol/L; uric acid: 556.0 μmol/L; high-density lipoprotein cholesterol: 0.61 mmol/L. Blood gas analysis (nasal cannula oxygen 4 L/minute): glucose: 10.50 mmol/L; pH: 7.44; partial pressure of carbon dioxide: 47.0 mmHg; partial pressure of oxygen: 64.0 mmHg. Biochemical tests indicated: Cl 98.50 mmol/L; Ca 1.07 mmol/L. Bedside ultrasound: fluid-filled anechoic areas visible at the axillary lines of the left and right hemithorax, between the 8th and 9th intercostal spaces, with maximum fluid depths of 86 mm and 122 mm. Electrocardiography showed complete left bundle branch block, P wave changes, and ST-T changes. Cardiac echocardiography: bilateral atrial enlargement with diminished contractility; moderate valvular regurgitation in all valves. Thyroid ultrasound: bilateral solid nodules in thyroid lobes, thyroid imaging reporting and data system category 3. Admission diagnosis: organic mental disorder; AHF, New York Heart Association class IV; bilateral pleural effusion; pulmonary edema; complete left bundle branch block; hypertension stage 2 (moderate risk); coronary atherosclerosis; hepatic impairment; chronic bronchitis with pulmonary emphysema; thyroid nodules; fatty liver; hyperuricemia; hypoproteinemia; hypochloremia.

### 2.2. Treatment and outcome

Upon admission, immediately initiate cardiac monitoring and administer oxygen via nasal cannula (flow rate 2.0 L/minute). Instruct the patient to remain strictly bedridden in a semi-recumbent position to reduce preload on the heart. Two intravenous accesses were established. Per physician orders, 10 mg nitroglycerin injection was administered via a microinfusion pump at 1.5ml/hour, with dosage dynamically adjusted based on blood pressure to reduce cardiac preload and afterload and improve coronary perfusion. Concurrently, 40 mg furosemide injection was administered as a bolus diuretic to alleviate pulmonary edema and systemic edema, with diuretic dosage adjusted daily according to urine output and edema severity. For bilateral massive pleural effusions, right-sided thoracentesis was performed under local anesthesia on the second day post-admission, draining 660 ml of yellowish pleural fluid. A chest tube was placed postoperatively with close monitoring of drainage characteristics, volume, and complications such as pneumothorax. Bedside ultrasound on the third postoperative day showed significant effusion reduction, leading to tube removal.

For organic mental disorders, multidisciplinary collaborative care was implemented alongside heart failure management.^[7,8]^ Daytime sessions included orientation training and psychological reassurance. At night, 3 mg of zopiclone tablets were administered orally per medical orders to improve sleep, stabilize delirium, prevent emotional agitation, and avoid exacerbating cardiac load due to nocturnal delirium. Strictly implement precise volume management using infusion pumps to control fluid rates, meticulously record 24-hour fluid balance, and maintain a daily negative fluid balance of 400 to 500 mL (actual 24-hour intake maintained around 2300 mL, output around 2000 mL). Strictly restrict sodium intake. Enhanced monitoring of electrolyte and blood gas analysis to maintain serum potassium and sodium within normal ranges; provided a high-protein diet and intravenous albumin supplementation to correct hypoproteinemia. The following therapeutic measures were concurrently implemented during the patient’s hospitalization: continuous bedside ultrasound for dynamic assessment of cardiac function and pleural effusion volume changes; strict aseptic technique and terminal disinfection to prevent nosocomial cross-infections, particularly respiratory and urinary tract infections; administration of antiplatelet therapy and circulatory-enhancing agents to prevent thrombus formation; implementation of bundled infection control measures throughout the entire hospitalization period.

After 16 days of comprehensive symptomatic treatment and precise nursing care, the patient’s symptoms of chest tightness and shortness of breath improved significantly. The patient was able to sleep in a supine position at night, and the moist rales at the bases of both lungs disappeared. Edema in both lower limbs and the face subsided markedly. A follow-up bedside ultrasound showed that the bilateral pleural effusions had largely resolved, and the ejection fraction had improved. The patient was alert, with restored orientation to time, place, and person. The patient was emotionally stable, with good appetite and sleep. Body weight had decreased from 65 kg at admission (including edema) to 53.8 kg (dry weight). Follow-up tests, including liver and kidney function, cardiac enzyme panels, and arterial blood gas analysis, showed significant improvement compared to admission. No cases of nosocomial cross-infection, pressure ulcers, or secondary infections occurred during hospitalization. At the 3-month post-discharge follow-up, the patient’s blood pressure remained stable at 130 to 140/80 to 90 mm Hg, with a heart rate of 70 to 80 beats per minute. The patient was adhering to the prescribed regimen of anti-heart failure and psychiatric medications, was largely self-sufficient in daily activities, and had not experienced any recurrence of AHF or exacerbation of psychiatric symptoms.

## 3. Management and nursing

**Figure 1.**
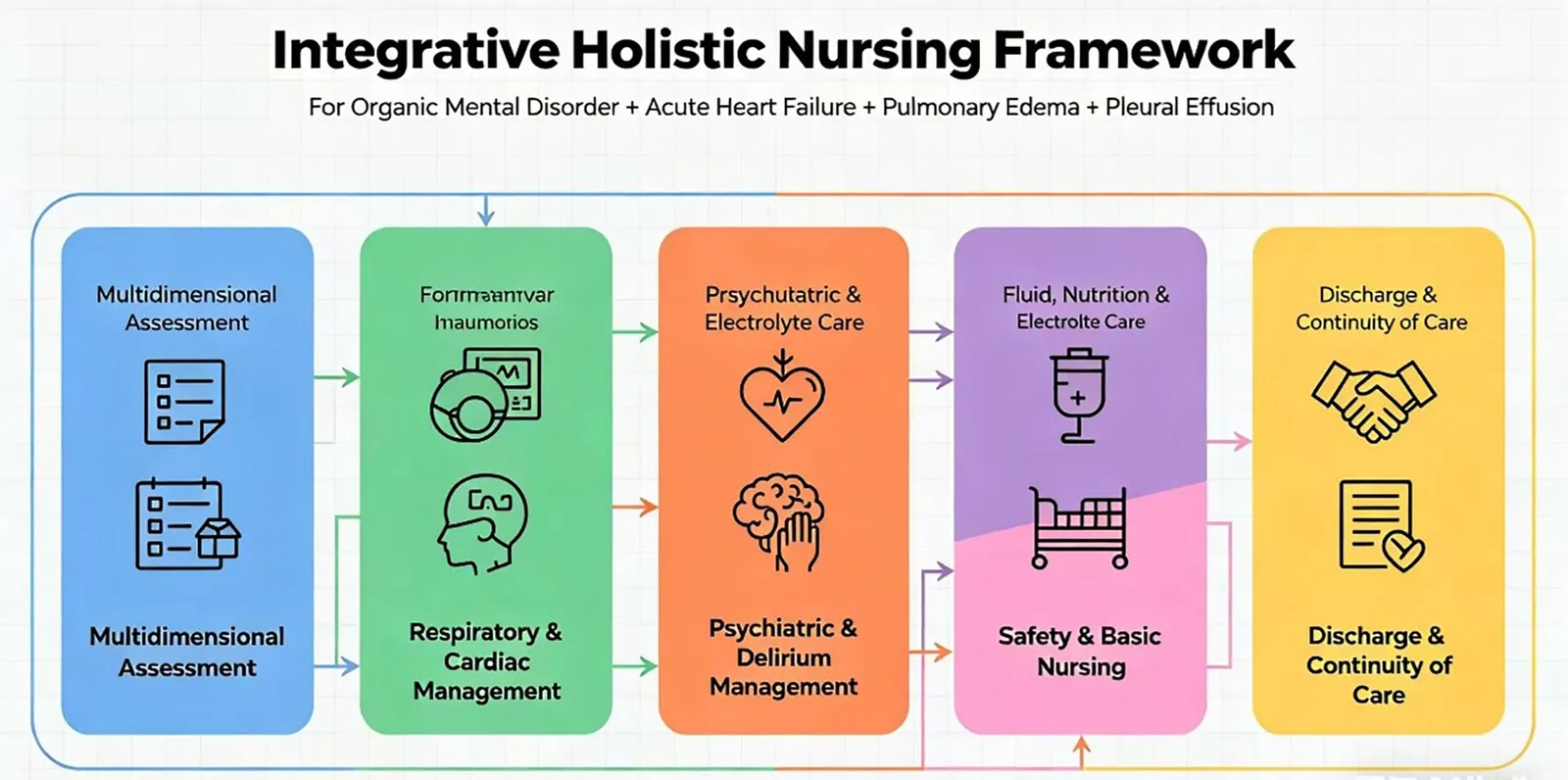
Integrative holistic nursing framework for a patient with organic mental disorder complicated byAHF, pulmonary edema, and pleural effusion. The framework comprises 5 sequential yet interrelated modules: multidimensional assessment (consciousness, respiration, circulation); respiratory & cardiac management (high-flow oxygen therapy, positional management, ultrasound-guided fluid adjustment); psychiatric & delirium management (daytime cognitive orientation, nighttime light sedation); fluid, nutrition & electrolyte care and safety & basic nursing (precise volume management, bundled infection control); and discharge & continuity of care. Arrows indicate the dynamic, bidirectional relationships between modules. This figure was created by the authors based on holistic nursing theory. AHF = acute heart failure.

Based on the patient’s complex conditions, we developed an integrative holistic nursing framework encompassing 5 interconnected dimensions: multidimensional assessment, respiratory and cardiac management, psychiatric and delirium management, fluid/nutrition/electrolyte care and safety/basic nursing, and discharge with continuity of care (Fig. 1). This figure was created by the authors based on the holistic nursing theory.^[5]^ Given the complex and critical nature of patients with organic mental disorders complicated by AHF, characterized by the rapid progression and mutual influence between psychiatric symptoms and cardiac dysfunction, our hospital has developed and implemented individualized precision nursing protocols.

### 3.1. Ward management

Upon admission, the psychiatric emergency response protocol was immediately activated to optimize ward functionality and resource allocation. The patient was placed in a resuscitation room equipped with comprehensive monitoring capabilities and facilitating observation, under closed-door management. Warning signs were installed throughout the ward, delineating clean, semi-contaminated, and contaminated zones while standardizing personnel movement routes. Hazardous items were removed from the room, protective equipment was installed, and safety barriers were established to prevent agitation, falls, and self-injury.^[9]^

Form joint psychiatric and general internal medicine nursing teams. Conduct training on critical care coordination and emergency management of psychiatric symptoms. Implement flexible scheduling to ensure adequate staffing during peak periods. Strictly enforce disinfection and isolation protocols, properly handle patient secretions and medical waste, and implement standard precautions.^[10]^ Establish visitation management systems, provide safety education to family members, and collaborate to maintain ward security and order.

### 3.2. Personalized precision care

Given the patient’s concurrent AHF, pulmonary edema, bilateral pleural effusions, organic mental disorder, hypoalbuminemia, electrolyte imbalance (hypochloremia), liver impairment, and sleep disturbance, establish a precision nursing system to simultaneously implement dual physical and psychological safety protocols for impulse control, fall prevention, and pressure ulcer prevention.

#### 3.2.1. Vital signs and condition monitoring

Upon admission, continuously monitor vital signs, electrocardiography, blood pressure, and blood oxygen saturation. Use disposable protective covers for monitor probes; dispose of them as medical waste after use. Upon admission, the patient presented with a blood pressure of 139/90 mm Hg and oxygen saturation of 60% (85% after oxygen administration). Immediately initiate the dedicated monitoring protocol for Type II respiratory failure. Administer low-flow oxygen via nasal cannula (1–2 L/minute) while dynamically reassessing arterial blood gas analysis, with particular focus on monitoring partial pressure of carbon dioxide changes to prevent carbon dioxide retention. Simultaneously, meticulously record 24-hour fluid balance, measure daily weight, and assess edema resolution (severe facial and bilateral lower limb edema gradually subsided). Remain vigilant for malignant arrhythmias due to complete left bundle branch block and early signs of acute left ventricular failure (orthopnea, jugular venous distension). Dynamic data logging provided the basis for adjusting diuretics, antihypertensive medications, and oxygen therapy protocols.^[11]^

#### 3.2.2. Respiratory care

Maintain airway patency. Oxygen therapy should be administered via nasal cannula at low-flow rates (1–2 L/minute) using dedicated equipment. Replace sterile distilled water in the humidifier bottle daily to prevent aggravated carbon dioxide retention from high-concentration oxygen therapy. Assist the patient in assuming a semi-recumbent or upright sitting position to reduce cardiac preload. Encourage the use of disposable tissues to cover the mouth and nose during spontaneous coughing to expel sputum. If sputum is viscous, administer nebulized inhalation therapy as prescribed. Following right-sided thoracentesis, closely monitor the drainage site for serous fluid, subcutaneous emphysema, and improvement in dyspnea, while remaining vigilant for tension pneumothorax. Increase nighttime rounds to observe for snoring and arousal from breathlessness, and instruct patients to sleep in a lateral position to improve obstructive hypoventilation.

#### 3.2.3. Fluid and nutrition management

Establish a strict 24-hour fluid intake and output monitoring system, controlling daily fluid intake (approximately 2300 ml) based on the principle of “output determines intake,” and accurately record hourly urine output and edema resolution^[12]^; administer furosemide and spironolactone diuretics as prescribed, dynamically monitor serum potassium and chloride levels, and promptly correct electrolyte imbalances with oral potassium chloride sustained-release tablets. For hypoalbuminemia (albumin 29.48 g/L), implement a high-protein, high-vitamin, easily digestible liquid/semiliquid diet with restricted sodium intake. Daily assess weight changes and nutritional status to ensure a negative fluid balance of 400 to 500 ml to promote resolution of pulmonary edema and systemic edema.

#### 3.2.4. Management of psychiatric symptoms and safety protection

For hallucinations, disorientation, and circadian rhythm disruption caused by organic mental disorders, implement 24-hour monitoring with bed rails to prevent falls. Remove hazardous items from the ward and maintain dry, nonslip flooring. Avoid arguing with patients about the content of their hallucinations; instead, stabilize their emotions through gentle guidance and distraction techniques. Minimize auditory and visual stimuli at night while increasing rounds to prevent falls or wandering during agitation episodes.^[13]^ Administer zopiclone and quetiapine fumarate as prescribed, monitoring sedation effects and adverse reactions such as orthostatic hypotension to ensure patient safety.

#### 3.2.5. Basic care

For patients with severe generalized edema, use air-cell mattresses and pressure-relieving dressings to protect bony prominences. Turn the patient every 2 hours and monitor skin integrity; promptly change sweat-soaked clothing to maintain bed unit dryness. Perform oral care 2 to 3 times daily to prevent mucosal ulcers and infections. Closely monitor puncture sites for bleeding or serous discharge. Maintain venous access with strict aseptic technique. Record 24-hour urine output and characteristics. Keep the perineal area clean to prevent related infections and complications.^[14,15]^

#### 3.2.6. Psychological and sleep care

Throughout the process, narrative care is employed to alleviate patients’ psychological stress while strictly safeguarding privacy. The psychiatric symptom care team dynamically adjusts intervention plans based on sleep monitoring data, closely observing the efficacy and adverse effects of sedative medications to prevent sleep disturbances from compromising immunity and bodily repair processes.^[16]^

### 3.3. Discharge and continuing care

Patients must meet the following discharge criteria: resolution of chest tightness and shortness of breath, resolution of generalized edema, ability to sleep supine at night, alleviation of psychiatric symptoms, and stable vital signs. Follow-up bedside ultrasound should show a significant reduction in pleural effusion and pericardial effusion, improved cardiac ejection fraction, and normalization of liver and kidney function, cardiac enzyme levels, and electrolyte levels. Discharge is approved following comprehensive assessment by the psychiatric expert panel.^[17]^ Prior to discharge, provide health education covering medication adherence (e.g., diuretics require monitoring of serum potassium and urine output; antihypertensives necessitate heart rate and blood pressure checks; antipsychotics require precautions against falls; antiplatelet agents demand vigilance for bleeding risks), self-monitoring protocols (daily recording of blood pressure, heart rate, weight, and lower limb edema), and key points for managing abnormal symptoms (identifying worsening dyspnea, nocturnal awakening due to chest tightness, visual hallucinations as recurrence signs). Assist with discharge procedures and handover. Develop a comprehensive health guidance plan tailored to the patient’s disease characteristics, rehabilitation needs, and home environment, covering disease awareness, medication adherence, self-monitoring, emergency management, and psychological adjustment. Establish a follow-up record. Schedule follow-up appointments at the specialty clinic at 1 week, 1 month, and 3 months post-discharge for blood counts, electrolytes, B-type natriuretic peptide, liver/kidney function tests, and cardiac ultrasound. Assign a dedicated staff member to periodically monitor the patient’s physical condition, medication adherence, and psychological status. A follow-up visit 3 months after discharge revealed that the patient was taking medication regularly as prescribed, blood pressure was well controlled, and there had been no recurrence of AHF or worsening of psychiatric symptoms.

## 4. Discussion

Elderly patients with organic mental disorders complicated by AHF, pulmonary edema, and pleural effusion present with complex and critical clinical manifestations. The mutual influence and vicious cycle between mental disorders and cardiac dysfunction pose significant challenges to clinical nursing. This patient exhibited multisystem dysfunction involving consciousness, respiration, and circulation, alongside psychiatric symptoms such as delirium, hallucinations, and disorientation. The rapid progression and insidious changes in the condition necessitated holistic, systematic interventions addressing physiological, psychological, social, and psychiatric dimensions. Based on the nursing practice summary for this case, we propose that the key nursing priorities for such complex cases include establishing a multidimensional assessment system grounded in holistic nursing theory; implementing personalized, precision care addressing physiological, psychological, social, and psychiatric dimensions; establishing an integrated care model through multidisciplinary collaboration; and enhancing discharge planning and continuity of care services.

First, holistic nursing theory emphasizes viewing the individual as a unified whole encompassing physiological, psychological, social, and spiritual dimensions, rather than merely a collection of diseases or symptoms.^[6]^ As a 67-year-old male, this patient exhibited coexisting organic mental disorders alongside AHF, pulmonary edema, and other physical conditions, demonstrating classic mind-body interaction. Traditional single-specialty nursing approaches struggle to address such complexity; an integrative perspective is essential. This requires incorporating cognitive function, emotional state, cardiopulmonary function, nutritional status, sleep patterns, and social support systems into a unified assessment framework. A randomized controlled trial by Heo et al^[6]^ in heart failure patients demonstrated that mindfulness-based interventions grounded in holistic care significantly improved anxiety, depression, and quality of life. These benefits extended beyond psychological dimensions to stabilize physiological indicators. In this nursing practice, we established a continuous 3-dimensional assessment system for consciousness, respiration, and circulation to detect early signs of deteriorating brain, heart, and lung interactions, enabling dynamic monitoring of the patient’s overall condition. This multidimensional monitoring focuses not only on physiological indicators like blood pressure, heart rate, and blood oxygen saturation but also incorporates psychological indicators such as level of consciousness, orientation, and emotional responses, embodying the holistic care principle of caring for the whole person.

Second, the comorbidity of organic mental disorders and heart failure is highly prevalent among elderly patients, yet cognitive impairment is often overlooked. A prospective cohort study by Agarwal et al^[1]^ revealed that unrecognized cognitive impairment is an independent risk factor for increased readmission rates in elderly heart failure patients. Patients with cognitive impairment exhibit diminished self-management abilities, reduced medication adherence, impaired symptom recognition, and compromised understanding of dietary control, leading to suboptimal treatment outcomes. This patient presented with pronounced disorientation and hallucinations upon admission, concurrent with acute left ventricular failure. While managing heart failure, the nursing team must also address treatment compliance challenges arising from psychiatric symptoms. We adopted a multidisciplinary collaborative approach, with psychiatric and general internal medicine nurses jointly developing a care plan. Daytime orientation and cognitive training enhanced the patient’s reality orientation, while mild sedation at night maintained sleep rhythms to prevent diurnal reversal from exacerbating cognitive disorientation. Pressler et al^[18]^ found that involving caregivers in managing patients with cognitive impairment significantly reduced 30-day readmission rates and improved self-care maintenance, consistent with our practice.

Third, symptom management and comfort care are integral components of holistic nursing practice, particularly during acute decompensation. Nurses must balance alleviating physical symptoms with preserving patient dignity. Upon admission, this patient presented with severe dyspnea, generalized edema, and sleep deprivation, experiencing profound distress. The nursing team employed high-flow oxygen therapy combined with positional management, dynamically adjusting fluid balance under bedside ultrasound guidance to rapidly alleviate pulmonary edema. Concurrently, thoracentesis was performed with close monitoring of drainage characteristics and respiratory improvement. Self-care capacity in heart failure patients directly influences disease prognosis, with cognitive function serving as its foundation. Currie et al^[19]^ systematic review found mild cognitive impairment significantly correlates with impaired self-care maintenance and monitoring abilities in heart failure patients. During this patient’s hospitalization, the nursing team employed simplified instructions, repeated reinforcement, and visual cues to progressively cultivate patient compliance. Kinnunen et al^[20]^ proposed a model of interaction among context, body, and environment, emphasizing holistic nursing practice encompassing physical care, situational sensitivity, and environmental design. This case created a safe healing environment by removing hazardous items from the ward and reducing nocturnal auditory and visual stimuli, exemplifying the environmental dimension of holistic care.

Fourth, discharge preparation and continuity of care are crucial manifestations of holistic nursing continuity. For elderly patients with chronic heart failure and psychiatric disorders, the quality of transitional care directly impacts readmission rates and long-term prognosis. This case established clear discharge criteria (including symptom resolution, pleural effusion resolution, stable psychiatric symptoms, and stable vital signs) confirmed through comprehensive assessment by a psychiatric expert panel. predischarge health education covered medication adherence, self-monitoring, and recognizing abnormal symptoms, with follow-up records established and regular monitoring scheduled. However, continuity of care for such patients remains inadequate. First, there is a lack of systematic tracking mechanisms and comprehensive health record monitoring systems, resulting in insufficient continuity for symptom management, medication adherence, and rehabilitation monitoring after discharge. Second, community nursing capacity is weak, and dynamic information synchronization with medical institutions is unattainable, resulting in gaps in home monitoring and health management after discharge. This case represents a single case report, limiting the generalizability of nursing experiences; the evaluation of holistic nursing intervention outcomes primarily relies on clinical observation and short-term follow-up, lacking standardized quantitative holistic nursing outcome indicators. Future research should conduct multicenter, large-sample cohort studies to develop holistic nursing quality assessment tools tailored for elderly patients with organic mental disorders and comorbid physical illnesses. This requires enhancing continuity of care systems, strengthening community chronic disease management capabilities, and establishing a 3-tiered collaborative mechanism involving hospitals, communities, and families.

In summary, the successful treatment of this elderly patient with organic mental disorder complicated by AHF, pulmonary edema, and pleural effusion validates the core value of holistic nursing in managing complex critically ill patients. By establishing a multidimensional assessment system, implementing individualized precision nursing, fostering multidisciplinary collaboration, enhancing symptom management and comfort care, integrating psychosocial-spiritual support, and improving continuity of care, we achieved the goal of holistic, continuous, and comprehensive nursing. This practice provides a referenceable standardized process and practical experience for holistic nursing in elderly patients with cardiovascular and cerebrovascular comorbidities, aligning with the contemporary shift in nursing from a disease-centered to a health-centered paradigm. Future efforts should further refine holistic nursing standards for patients with complex comorbidities, strengthen continuity between hospital and community care, and truly achieve high-quality, patient-centered holistic care.

## Author contributions

**Data curation:** Guohui Jiao, Yongcai Wu.

**Investigation:** Guohui Jiao, Yongcai Wu, Shanshan Zhang.

**Supervision:** Haiyan Wang, Xiaoli Du.

**Visualization:** Haiyan Wang, Xiaoli Du.

**Writing – original draft:** Shanshan Zhang.

**Writing – review & editing:** Guohui Jiao, Yongcai Wu.
